# Genome Sequence of *Staphylococcus aureus* Strain HUK16, Isolated from Hexachlorocyclohexane-Contaminated Soil

**DOI:** 10.1128/genomeA.00274-16

**Published:** 2016-04-14

**Authors:** Cyrielle Gasc, Jean-Yves Richard, Pierre Peyret

**Affiliations:** a EA 4678 CIDAM, Université d’Auvergne, Clermont-Ferrand, France; b SITA Remediation, Suez, Meyzieu, France

## Abstract

*Staphylococcus aureus* strain HUK16 has been isolated from hexachlorocyclohexane (HCH)-long-term contaminated soil. The genome of strain HUK16 was sequenced to understand the genetic basis of its adaptation to HCH and to find the potential metabolic pathways allowing it to degrade the pesticide. Here, we report the annotated draft genome sequence (~2.7 Mbp) of this strain.

## GENOME ANNOUNCEMENT

The overuse of hexachlorocyclohexane (HCH) as an insecticide around the world to control agricultural pests has led to its large-scale dissemination in the environment (1). Therefore, because of its toxicity and persistence in agricultural and industrial sites, its use is now restricted or banned in many countries (2). The study of indigenous microbial communities of contaminated soils is necessary to evaluate the possibility of establishing a bioremediation approach to clean up these polluted sites (3). In this context, an aerobic HCH-supplemented liquid cultivation of microorganisms from soil contaminated with HCH in an ancient chemical factory (Huningue, France) has been carried out, and subsequent repeated seeding of individual colonies has led to the isolation of the *Staphylococcus aureus* strain HUK16 (SITA Remediation, Suez, France). The capability of the strain to grow in minimal salt medium containing HCH as the sole carbon source suggested its capability to degrade the molecule for its metabolism.

Thus, to better understand how strain HUK16 is adapted to HCH and which degradation pathway it possibly possesses, the genomic DNA of the strain was sequenced by use of an Illumina HiSeq 2500 platform (CASAVA version 1.8.2). The shotgun sequencing generated 4,195,151 high-quality paired-end reads, which were quality trimmed with Trimmomatic (version 0.32) (4) and assembled *de novo* using Velvet (version 1.2.10) (5). The draft genome consists of 74 contigs, with an *N*_50_ length of 120 kbp, an average length of 366.93 kbp, and a max length of 502.63 kbp. The total contig sequence represents 2,715,311 bp and has a G+C content of 32.78%. The validated genome assembly was annotated using the NCBI Prokaryotic Genome Annotation Pipeline (PGAP) (http://www.ncbi.nlm.nih.gov/genome/annotation_prok/). A total of 2,590 protein-coding genes and 90 pseudogenes were identified.

Different pathways for HCH degradation have been characterized and involve the *linA* to *linJ* genes (6). All characterized degrading bacterial species described to date possess these enzymes. To understand how strain HUK16 degrades HCH, a BLAST search within its genome using the *lin* gene sequences of *Sphingobium indicum* B90A (accession no. AJXQ00000000) was performed. None of these genes or their homologues, and no other gene likely to be involved in HCH degradation, have been identified. Thus, the combination of these results and cultural observations seem to indicate that *S. aureus* strain HUK16 degrades HCH using metabolic pathways and enzymes not previously described in the literature, as suggested by previous studies (7).

### Nucleotide sequence accession numbers.

This whole-genome shotgun project has been deposited in DDBJ/ENA/GenBank under the accession no. LSMV00000000. The version described in this paper is the first version, LSMV00000000.1.
